# Primary stent implantation for bilateral spontaneous cervical ICA dissections with hypoperfusion after 72 h from onset: a case report

**DOI:** 10.1186/s42155-022-00318-x

**Published:** 2022-08-13

**Authors:** Yijie Chen, Ningyuan Zhang, Yigang Chen, Xu Zheng, Xing Jin, Jinhua Zhang

**Affiliations:** 1 Department of Neurology, Sir Run Run Shaw Hospital, Zhejiang University School of Medicine, No.3, East Qingchun Road, Hangzhou, China; 2 Department of Neurology, the First Hospital of Tongxiang, Jiaochang Road 1918, Tongxiang, Zhejiang China

**Keywords:** Spontaneous dissection, Internal carotid artery, Endovascular treatment, Stenting, Acute ischemic stroke

## Abstract

**Background:**

Spontaneous cervical internal carotid artery dissection (cICAD) is a common cause of stroke in young adults. Endovascular therapy is an indispensable treatment for cICAD in some cases, but it faces great challenges.

**Case presentation:**

A bilateral spontaneous cICADs with hypoperfusion-related AIS after 72 h from the onset was presented herein. The patient responded well to primary Solitaire stent detachment at the critical flow-limiting site.

**Conclusions:**

Primary stent implantation at the critical flow-limiting site rather than covering the entire dissection may be a therapeutic option in spontaneous cICAD complicated with cerebral hypoperfusion. The Solitaire stent may be a good choice at the acute and subacute stages of cICAD.

## Background

Spontaneous cervical internal carotid artery dissection (cICAD) is a relatively common cause of stroke in young adults (Engelter et al. 2015). It causes acute ischemic stroke (AIS) through arterial-arterial embolism, hypoperfusion, and mixed mechanisms. Previous studies show that 85% of spontaneous cICAD-related AIS occurs from A-A embolism, and 15% of AIS occurs from hypoperfusion or mixed mechanisms (Morel et al. 2012). Endovascular treatment (EVT) restores the vessel caliber and normal circulation, repairing the arterial defect and thus preventing the formation of emboli. Previous studies show that EVT is crucial for those patients who do not respond to antithrombotic treatment, have clinical deterioration due to hypoperfusion, or experience acute occlusion in an intracranial large vessel (Engelter et al. 2015; Moon et al. 2017; Engelter et al. 2021). However, the EVT brings challenges: 1) to secure true lumen during the whole procedure; 2) to prevent downstream embolism from thrombus migration; 3) multiple stents are serially implanted using partially overlapping techniques to reconstruct the entire dissected segment, creating a restriction for the type of stent for deployment (Cohen et al. 2003; Ansari et al. 2017). Currently, the exact procedure for EVT remains ambiguous. Herein, we presented a case of primary stent implantation to repair a critical flow-limiting cavity to improve cerebral perfusion caused by a spontaneous bilateral cICAD and hypoperfusion-related AIS after 72 h of onset and achieved a good outcome.

## Case presentation

**Fig. 1:**
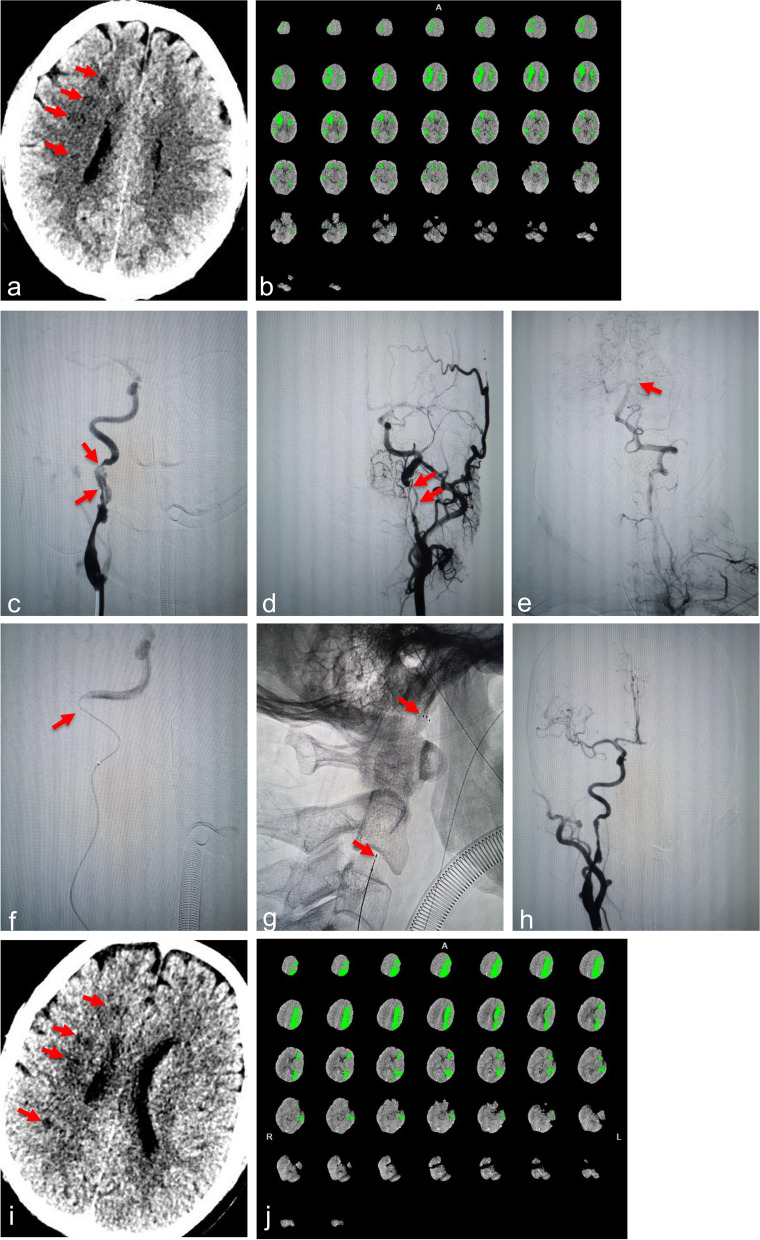
The first procedure. **a-b** On Sep 2, 2020, emergent head NCCT revealed new infarctions in the right internal watershed area. (a. red arrows), head CTP showed an ischemic penumbra of 100.3 mL in the bilateral ICAs (green area). **c-h** Emergent EVT on Sep 3, 2020: Preprocedural angiogram showed long stenosis with a double lumen sign, distal to the right carotid bulb (c. red arrows indicated a flow-limiting segment), string-like stenosis distal to the left carotid bulb (d. red arrows indicated a flow-limiting segment) with an opening into left PCom A (e. red arrow); The microcatheter was in the true lumen, which was confirmed by post-lesion angiography (f. red arrow); A 6 × 30 mm Solitaire FR stent was temporarily deployed in the key flow-limiting segment through a microcatheter (g. red arrows indicated the distal and proximal markers of the stent); After the stent release, angiogram showed that the stenosis of the lesion was reduced, the double-lumen sign disappeared, the anterior blood flow was significantly improved, and a small amount of compensation was made to the left anterior circulation through ACom A (h). **i-j** On Sep 4, 2020, repeated head NCCT revealed more pronounced infarctions than the pre-procedure status (red arrows), head CTP showed that the perfusion of the blood supply area of the right ICA was recovered, and the penumbra of the blood supply area of left ICA was enlarged to 114.2 mL, compared with that before the procedure (green area)

**Fig. 2:**
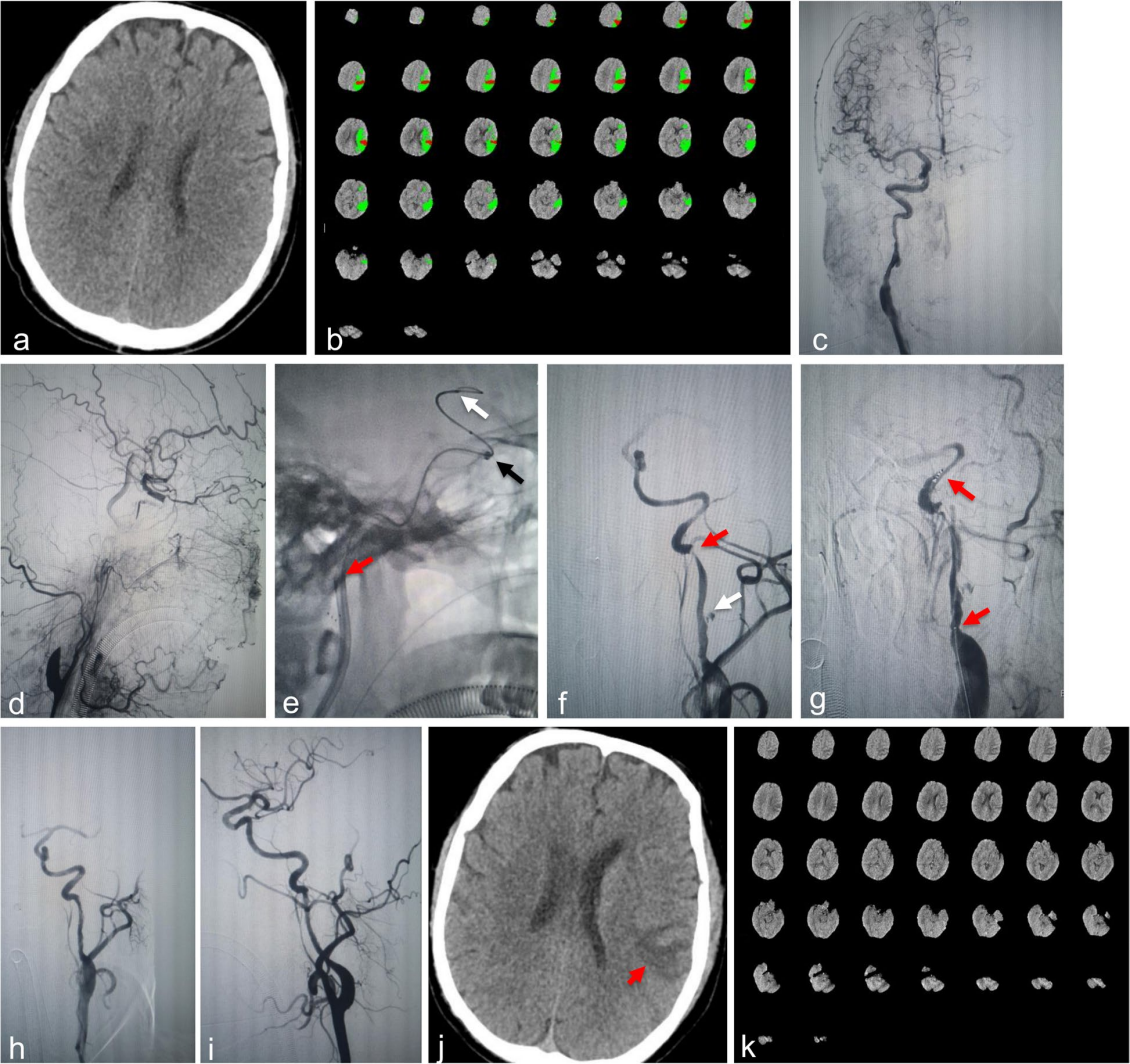
The second procedure. **a-b** On Sep 9, 2020, emergent head NCCT showed no new lesion in the left hemisphere, and head CTP revealed a new core infarct of 18.2 mL in the left frontal lobe with an ischemic penumbral area of 100.4 mL in the left ICA supply area. **c-i** On Sep 9, 2020, an emergent EVT was performed. Preprocedural angiogram showed that the right ICA remained patent with residual moderate-to-severe stenosis and dissecting aneurysm and compensation to the left ACA and MCA via the ACom A (c), the left ICA was occluded distal to the bulb and manifested a flame sign with a refluxed flow to the C4 segment via the ophthalmic artery in the distal end (d); the position of the microcatheter tip (e. white arrow), the position of the CAT6 tip (e. black arrow), the position of the 8F guide catheter tip (e. red arrow); Applying with SCP technique, the left ICA was revascularized with a residual dissecting aneurysm (f. white arrow) and a red arrow indicated the key flow-limiting stenosis (f); red arrows indicated the distal and proximal markers of the 6 × 30 mm Solitaire FR stent (g); the P-A and oblique angiogram after stent detachment showed that the stenosis was relieved and the dissecting aneurysm disappeared (h-i). **j-k** On Sep 14, 2020, repeated head NCCT showed that the new core infarct appeared in the left frontal lobe, and head CTP suggested that the perfusion of bilateral ICAs blood supply areas returned to normal

A 46-year-old man with a history of hypertension, smoking, and alcohol use experienced a stroke on waking up with left limb weakness (4/5) and slurring speech on August 30, 2020. He was diagnosed to have a right internal carotid artery (ICA) territory infarct based on non-contrast computed tomography (NCCT) at local hospital. Despite antiplatelet therapy with aspirin 100 mg/d and clopidogrel 75 mg/d, over the next 3 days, left limb weakness progressively worsened (2/5), accompanied by somnolence (GCS 3 + 5 + 6). Therefore, he was transferred to our hospital on September 2, 2020. The Glasgow Coma Scale (GCS) score was 13/15. The head NCCT revealed new infarcts in the right internal watershed area and CT perfusion (CTP) showed a 100.3 mL mismatch of bilateral cerebral hemispheres, indicating that a considerable penumbral region was present in the supply area of each internal carotid artery (ICA) (Fig. 1a and b). Emergent EVT was performed under general anesthesia. The patient received IV heparin (50 U/Kg) before the surgical procedure. Preprocedural angiogram showed a double-lumen sign in the ascending segment of the right ICA with severe stenosis of the true lumen and linear stenosis in the ascending segment of the left ICA, suggesting dissections (Fig. 1c and d). The anterior communicating artery (ACom A) and the right posterior communicating artery (PCom A) were not open, and the left posterior cerebral artery (PCA) mildly compensated the left middle cerebral artery (MCA) through ipsilateral PCom A (Fig. 1e). Because we judged that the right ICA was the main vessel responsible for stroke, and worried about the risk of hyperperfusion-related bleeding in the simultaneous treatment of bilateral ICAs, we decided to treat right ICA first. An 8F Envoy guide catheter (Johnson & Johnson Co. Ltd., New Brunswick, NJ, USA) and 5F MPA catheter were introduced into the distal common carotid artery (CCA) by applying a coaxial technique. After traversing the true lumen with a Trevo Pro 18 microcatheter over a Synchro-2 microwire (Stryker Corp., Fremont, CA, USA), a Solitaire FR 6 × 30 mm stent-retriever (Medtronic Inc., Wexford, PA, USA) was temporarily deployed at the key flow-limiting site (Fig. 1g). An angiogram showed an image of a patent true lumen with a significantly reduced false lumen. After observation of 30 min, blood flow was maintained well, and ipsilateral anterior cerebral artery (ACA) compensated left ACA through ACom A. Thus, the Solitaire stent was detached (Fig. 1h). After recovery from anesthesia, the muscle strength of left limb was improved from grade 2/5 to grade 3/5, but the somnolence was not improved with a 13/15 GCS. Repeated CTP revealed that the perfusion of the right anterior circulation recovered, but a large area of hypoperfusion in the left anterior circulation was still seen (Fig. 1i and j). Due to concerns about clopidogrel resistance, the dual antiplatelet regimen was adjusted to aspirin 100 mg/d and ticagrelor 90 mg twice daily. On the 6th day after the procedure, the patient suffered from a generalized seizure and became stupor with a 9/15 GCS. Further, the muscle strength of right limb was decreased to grade 2/5. But no new lesions were found on the emergent head NCCT. Nevertheless, head CTP showed a new core infarction of 18.2 mL in the left frontal lobe with a penumbra of 100.4 mL in the left anterior circulation (Figs. 2a and b). Then, an emergent EVT was performed again. The angiogram revealed that the right ICA was patent with an inadequate compensation to the left anterior circulation via ACom A, and left ICA was occluded (Figs. 2c and d). A triaxial assembly including an 8F Mach1 guide catheter (Boston Scientific, Marlborough, MA, USA), AXS Catalyst 6 (Stryker Corp.), and a Trevo Pro 18 microcatheter over a Synchro-2 microwire were navigated through the left dissected segment (Fig. 2e). Subsequently, the Catalyst 6 and guide catheter were successively withdrawn to the beginning of the ICA under continuous negative pressure application, namely the simple catheter-passing (SCP) technique. Several dark red emboli were captured by Catalyst 6. A repeated angiogram showed that the left ICA was successfully recanalized and the structure of the dissection was fully revealed (Fig. 2f). After traversing the true lumen with the Pro 18 microcatheter over a Synchro-2 microwire, a Solitaire FR 6 × 30 mm stent-retriever was temporarily deployed at the key flow-limiting site (Fig. 2g). A subsequent angiogram showed that the antegrade blood flow was significantly improved and the dissecting aneurysm disappeared. After observation of 30 min, the Solitaire stent was detached. After recovery from anesthesia, the patient's consciousness became clear with a 15/15 GCS, the tracheal intubation was removed on the following day, and the muscle strength of four limbs was significantly improved to grade 4/5. A repeated head NCCT showed infarction in the left frontal lobe, but repeated head CTP showed that the cerebral perfusion of bilateral anterior circulations was recovered (Figs. 2j and k).

**Fig. 3:**
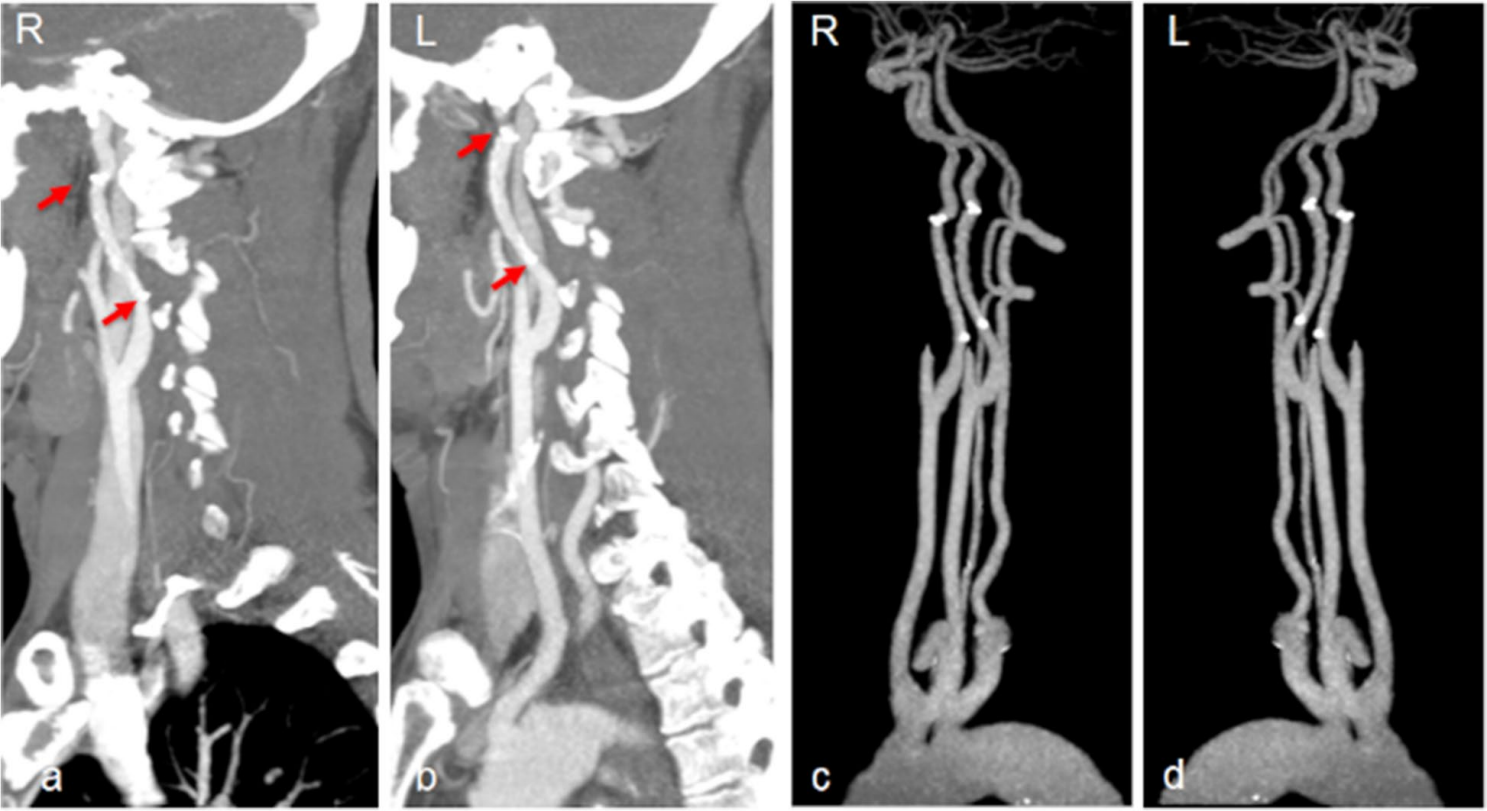
Follow-up images. **a-b** Follow-up CTA at three months showed that bilateral ICAs were repaired well (Nov 26, 2020); **c-d** Follow-up CTA at 15 months showed that bilateral ICAs remained patent and no dissection relapsed (Dec 13, 2021)

After 3 months of dual antiplatelet therapy, another 3 months of aspirin single antiplatelet therapy was followed. The modified Rankins Scale score (mRS) was 1 at the 90-day follow-up. Follow-up neck CTA at three months showed no residual lesion in both ICAs. No relapse of cerebral ischemic events during the 15-month follow-up occurred. A recent neck CTA showed that both ICAs remained patent without relapse of dissection (Fig. 3).

## Discussion

This was a typical case of bilateral spontaneous cICAD complicated with bi-hemispheric hypoperfusion that responded well to primary stent implantation at the critical flow-limiting site rather than covering the entire dissection. The lesions of cICAD are usually long segmented and the usual procedure of EVT uses a partial overlap technique to implant multiple stents in tandem to reconstruct the entire dissected segment (Cohen et al. 2003; Ansari et al. 2017). The advantages of this approach are: 1) to avoid residual dissection and then prevent secondary procedures in the future; 2) to cover the damaged intima to reduce the risk of thrombosis and thus lessen the chance of A-A embolism. The disadvantages of this approach are: 1) The implantation of multiple stents is probably unnecessary because cICADs have a strong self-healing ability. According to previous reports, the complete and hemodynamically significant (< 50% stenosis) recanalization rates of initial occlusion or high-grade stenosis in dissected carotid arteries are 60 to 67% of all cases within 6 months after the onset and the recanalization rate is at least 6.8% after 6 months of the onset (Sengelhoff et al. 2008; Nedeltchev et al. 2009; Baracchini et al. 2010); 2) The implantation of multiple stents increases the risk of acute or subacute in-stent thrombosis and increases the likelihood of mid- and long-term in-stent-restenosis; 3) The cost is high and the requirements for these types of stents are high. The present case found that primary stent implantation at critical flow-limiting site may be a simple and effective technique. Since the goal of emergent EVT was to improve blood flow rather than morphological perfection, balloon dilatation was unnecessary to achieve very low residual stenosis in the case. In addition, we were also concerned about the risk of distal embolization due to extrusion of the thrombus from the incision during balloon dilatation. Moreover, a relatively high residual stenosis reduces the risk of postprocedural hyperperfusion-related bleeding. In the present case, following improvement of antegrade blood flow of ICAs and under dual antiplatelet therapy, no downstream embolism was relapsed, and the dissected lesions uncovered by the stent were also restored by themselves.

A very challenging task of EVT in cICAD is choosing the type of stent for deployment. Based on previous reports, specific types of intracranial/carotid self-expanding stents might be good choice (Moon et al. 2017; Ansari et al. 2017; Sedat et al. 2003; Ishigami and Ota 2019). The main drawback of balloon expandable stents is the poor apposition to the vessel wall, especially after dissolution of the intramural hematoma. This goes along with delayed and frequently incomplete endothelialization. Additionally, there are reports of successful treatment of iatrogenic and spontaneous cICAD within 6 h of onset by direct detachment of the Solitaire stent (To et al. 2013; Shi et al. 2018). The lesions responded well because they were at the hyperacute stage allowing a relatively low radial force stent (Solitaire) to squeeze the intramural hematoma and regain the diameter of the true lumen. But there have been concerns that if the lesions become chronic or relapsing, an ongoing process of healing/further dissection takes over creating fibrotic bands that maybe not that amenable to be treated by low radial force stents. This is the reason why large parent vessel flow diversion implants like surpass streamliner can also recover the true lumen and assure a long-term vessel remodeling. The present case provided the first illustration that the Solitaire stent maybe still a good choice for spontaneous cICAD beyond 3 and 10 days after the onset. In addition, its characteristics of very low vessel wall coverage and good wall apposition are less thrombogenic (Krischek et al. 2011), so there is no need to worry too much about the risk of acute in-stent thrombosis. In the case of limited device selection, it is also of practical significance to make maximal use of Solitaire stent in China (Shi et al. 2018).

Furthermore, the present case taught us a lesson that we should be more active in intervening with the cICAD with cerebral hypoperfusion. If the left ICA lesion was treated earlier, the patient may have avoided the second ischemic event. There were reports on successful treatment of bilateral cICADs with one procedure (Sedat et al. 2003; Ishigami and Ota 2019).

## Conclusions

The present case demonstrated that primary stent placement at the critical flow-limiting site rather than covering the entire dissection may be a therapeutic option in spontaneous cICAD complicated with cerebral hypoperfusion, and the Solitaire stent may be a good choice at the acute and subacute stages of cICAD. Moreover, we should be more active in intervening with EVT in spontaneous cICAD with hypoperfusion.

## Data Availability

The datasets used and/or analyzed for the current report are available from the corresponding author upon a reasonable request. All data generated or analyzed for this study are included in this published article.

## Abbreviations

cICAD: Cervical internal carotid artery dissection
AIS: Acute ischemic stroke
EVT: Endovascular treatment
NCCT: Non-contrast computed tomography
CTP: Computed tomography perfusion
ACom A: Anterior communicating artery
PCom A: Posterior communicating artery
SCP: Simple catheter-passing
PCA: Posterior cerebral artery
MCA: Middle cerebral artery
mRS: Modified Rankins Scale score
